# Dynamic Contrast Enhanced Study in Multiparametric Examination of the Prostate—Can We Make Better Use of It?

**DOI:** 10.3390/tomography8030124

**Published:** 2022-06-09

**Authors:** Silva Guljaš, Mirta Benšić, Zdravka Krivdić Dupan, Oliver Pavlović, Vinko Krajina, Deni Pavoković, Petra Šmit Takač, Matija Hranić, Tamer Salha

**Affiliations:** 1 Clinical Department of Radiology, University Hospital Centre, 31000 Osijek, Croatia; zdravka.krivdic@gmail.com (Z.K.D.); matijahranic@gmail.com (M.H.); 2 Department of Mathematics, Josip Juraj Strossmayer University of Osijek, 31000 Osijek, Croatia; mirta@mathos.hr; 3 Department of Radiology, Faculty of Medicine, Josip Juraj Strossmayer University of Osijek, 31000 Osijek, Croatia; tsalha007@gmail.com; 4 Department of Urology, University Hospital Centre Osijek, 31000 Osijek, Croatia; pavlovic.oliver@kbco.hr (O.P.); krajina.vinko@gmail.com (V.K.); deni.pavokovic@gmail.com (D.P.); 5 Clinical Department of Surgery, Osijek University Hospital Centre, 31000 Osijek, Croatia; psmit292@gmail.com; 6 Department of Teleradiology and Artificial Intelligence, Health Centre Osijek-Baranja County, 31000 Osijek, Croatia; 7 Faculty of Dental Medicine and Health, Josip Juraj Strossmayer University of Osijek, 31000 Osijek, Croatia

**Keywords:** magnetic resonance imaging, muscle, perfusion, permeability, prostate cancer

## Abstract

We sought to investigate whether quantitative parameters from a dynamic contrast-enhanced study can be used to differentiate cancer from normal tissue and to determine a cut-off value of specific parameters that can predict malignancy more accurately, compared to the obturator internus muscle as a reference tissue. This retrospective study included 56 patients with biopsy proven prostate cancer (PCa) after multiparametric magnetic resonance imaging (mpMRI), with a total of 70 lesions; 39 were located in the peripheral zone, and 31 in the transition zone. The quantitative parameters for all patients were calculated in the detected lesion, morphologically normal prostate tissue and the obturator internus muscle. Increase in the K_trans_ value was determined in lesion-to-muscle ratio by 3.974368, which is a cut-off value to differentiate between prostate cancer and normal prostate tissue, with specificity of 72.86% and sensitivity of 91.43%. We introduced a model to detect prostate cancer that combines K_trans_ lesion-to-muscle ratio value and iAUC lesion-to-muscle ratio value, which is of higher accuracy compared to individual variables. Based on this model, we identified the optimal cut-off value with 100% sensitivity and 64.28% specificity. The use of quantitative DCE pharmacokinetic parameters compared to the obturator internus muscle as reference tissue leads to higher diagnostic accuracy for prostate cancer detection.

## 1. Introduction

Multiparametric magnetic resonance imaging (mpMRI) has become an increasingly used method for detecting clinically significant prostate cancer and navigating MRI and Transrectal ultrasound (TRUS) guided biopsy [1]. Also, it is used in the selection of patients for active surveillance and monitoring patients already on active surveillance [2].

Sequences included in mpMRI can be classified as anatomical high-resolution sequences—T1 and T2 weighted (T1W, T2W), and functional sequences—diffusion-weighted imaging (DWI), dynamic contrast-enhanced (DCE) MRI, and magnetic resonance spectroscopic imaging (MRSI), although MRSI is nowadays rarely in use [3]. T1W sequence is used to detect post-biopsy haemorrhage, pelvic lymphadenopathy and bone metastasis [4]. T2W is the main anatomical sequence providing differentiation of the peripheral zone from the transitional and central zone, detecting lesions and potential extracapsular extension of tumour or seminal vesicle invasion [5]. The main functional sequence is DWI and its apparent diffusion coefficient map (ADC), which provides information on tumour cellularity and tumour aggressiveness [6,7,8].

Lastly, DCE MRI is a functional imaging method that requires intravenous administration of gadolinium-based contrast agents and gives information regarding tumour vascularity and capillary permeability [9]. Some previous studies have found DCE to have higher accuracy in tumour localization compared to T2W or spectroscopic imaging [10,11]. Currently, it is used together with T2W and DWI to determine the category or in cases when findings are indeterminate or technical image quality is suboptimal due to motion or magnetic field distortion, mainly in the peripheral zone. DCE images can be interpreted using the qualitative, semi-quantitative and quantitative method [12]. Qualitative method includes visual detection of early and intensive enhancement followed by rapid washout in lesion compared to normal tissue. Semi-quantitative method generates time-intensity curves (type 1, 2 and 3) based on calculated parameters—time of contrast uptake, peak enhancement and washout gradient [13]. The final shape of the curve depends on the quality of the injection and parameter sequence. Inconsistencies in those parameters as well as the influence of tissue characteristics limit its reproducibility [14,15]. Also, it does not give any information regarding physiological and microvascular characteristics [1,9]. Finally, quantitative method uses the pharmacokinetic Tofts model to assess contrast concentration within the tissue. It is based on calculating transfer rate constants K_trans_ (measure of capillary permeability, efflux of contrast agent to extravascular extracellular space), K_ep_ (efflux of contrast agent back to plasma), V_e_ (leakage space, extracellular extravascular compartment volume) [16,17]. So, by observing the movement of contrast medium between intra- and extravascular space, information about tumour angiogenesis is collected [17].

This method takes into account which contrast media is used. Studies have shown that there is a difference in relative enhancement depending on the used contrast media, even when equimolar doses are applied at the same injection rate, but it does not influence pharmacokinetic parameters [18].

Qualitative and semi-quantitative methods are mostly used in everyday practice and in Prostate Imaging Reporting and Data System (PI-RADS) classification, due to their availability and simplicity [19], while quantitative method is less frequently used because it is more complex and requires special software [3].

Definitive diagnosis cannot be made based on one of these imaging techniques since morhphological and functional properties of cancer can overlap with benign conditions. Prostate mpMRI has high negative predictive value, but it also has too high false positive rate [20,21,22]. Therefore, we believe there is a need to increase specificity of the examination. In this study we have focused on the DCE MRI quantitative method as a very promising technique, but currently with insufficient data confirming its usefulness in the differentiation of benign conditions from clinically significant cancer.

Some previous studies have investigated the potential role of pharmacokinetic parameters in increasing the specificity and sensitivity of prostate mpMRI, especially K_trans_ and suggested cut-off values [23,24,25]. The limitations of these studies in determining universal cut-off value included limited reproducibility due to various factors, like different MRI scanners and DCE protocols [23,26]. PIRADS v.2 simplified DCE study analysis which led to improved interreader agreement in comparison to semi-quantitative analysis. However, there is still space for improvement and better usability of the DCE study [27].

The purpose of our study was to investigate whether quantitative DCE study can be used to differentiate cancer from normal tissue and to determine cut-off value of specific pharmacokinetic permeability parameters that predict malignancy more accurately by comparing them to the obturator internus muscle, which can serve as a reference tissue. It has less heterogenicity compared to prostate tissue and has arterial blood supply from a branch of the internal iliac artery.

## 2. Materials and Methods

### 2.1. Study Population

This retrospective study included a total of 190 patients who underwent prostate mpMRI at the University Hospital Centre Osijek, Department of Diagnostic and Interventional Radiology, from 2016 to 2021. The inclusion criteria were biopsy or prostatectomy proven prostate cancer (PCa), between 5 mm and 18 mm in size, after mpMRI detected organ confined unilateral lesions classified as PI-RADS 3, 4 or 5 in order to have a contralateral mirror area that is lesion free.

Inclusion criteria for the negative control group of healthy patients was a previously performed mpMRI scan classified as PI-RADS 1 or 2, stable Prostate-Specific Antigen (PSA) level, without suspicious digitorectal examination findings, previous biopsy negative for cancer. Patients that have PI-RADS 1 or 2 do not undergo biopsy after examination, and they only have a follow-up in PSA level monitoring.

Exclusion criteria were chronic kidney failure since intravenous contrast application was contraindicated in these patients (1), biopsy negative for PCa after mpMRI classified as PI-RADS 3, 4 or 5 or unavailable biopsy findings (37), previous treatment for prostate cancer (1) and inadequate or incomplete mpMRI exam (2). The final study sample included 56 prostate cancer patients with a total of 70 lesions. The final sample of the healthy control group was 54 patients.

The targeted biopsy and prostatectomy were performed by different urologists in several hospitals in Croatia. Some hospitals use MR/US fusion biopsy and same have targeted biopsy based on sector map and standardised radiology report.

This study was approved by the Ethical Committee at the University Hospital Centre Osijek.

### 2.2. MR Technique

All MRI exams were performed on Simens Magnet Skyra 3T MRI System (Simens AG, Healthcare Sector, Munich, Germany) using the American College of Radiology (ACR) recommended protocol for prostate MRI. Endorectal coil was not used. Standard protocol included turbo spin-echo T2W in the sagital, axial and coronal plane (voxel size 0.6 × 0.6 × 3.5 mm; field of view 200 mm; slice thickness 3.5 mm; time of repetition 7500.0 ms; time of echo 101 ms; flip angle 160°), turbo spin-echo T1 weighted images in the axial plane (voxel size 0.4 × 0.4 × 3.5 mm; field of view 200 mm; slice thickness 3.5 mm; time of repetition 700.0 ms; time of echo 12 ms; flip angle 160°), turbo spin-echo T1W for detecting lymphadenopathy in the axial plane (voxel size 0.6 × 0.6 × 5.0 mm; field of view 325 mm; slice thickness 5.0 mm; time of repetition 1120.0 ms; time of echo 13 ms; flip angle 150°), echo planar DWI (b50/700/1400 s/mm2: voxel size 1.9 × 1.9 × 4.0 mm; field of view 220 mm; slice thickness 4 mm; time of repetition 4500 ms; time of echo 61.0 ms; EPI factor 114) and ADC maps in the axial plane, B1 maps for T1 mapping (voxel size 5.9 × 5.9 × 5.0 mm; field of view 380 mm; slice thickness 5 mm; time of repetition 3480.0 ms; time of echo 1.97 ms; flip angle 8°) T1 sequence with multiple flip angle for T1 mapping (voxel size 0.7 × 0.7 × 2.0 mm; field of view 260 mm; slice thickness 2 mm; time of repetition 5.00 ms; time of echo 1.71 ms; flip angle 1–2°; flip angle 2–10°) and DCE study in axial plane(voxel size 1.4 × 1.4 × 3.5 mm; field of view 260 mm; slice thickness 3.5 mm; time of repetition 5.00 ms; time of echo 1.77 ms; flip angle 15°; number of measurements 35).

DCE MRI was performed using T1 VIBE axial dynamic sequence after injecting 0.2 mL/kg of gadoterate meglumine. Contrast was administrated at 4 mL/s with an automatic injector followed by 20 mL saline at the same injection rate. The pre-contrast and the post-contrast dynamic images were positioned at the same level and transverse images in the same orientation. Quantitative Imaging Biomarkers Alliance (QIBA) group recommendations were followed whenever possible [28].

### 2.3. Image Analysis

Software used for image analysis was Siemens syngo, via WebViewer v2018.1.5.0.

All MRI exams were analysed by two radiologists, with 18 and 6 years of work experience, respectively, in compliance with the PI-RADS v2.1 score.

Pharmacokinetic parameters K_trans_, K_ep_, initial Area Under Curve (iAUC) and V_e,_ were obtained in this quantitative study using the Tofts model. The population based arterial input function (AIF) was calculated.

For every patient we manually drew the region of interest (ROI) on DCE images using the ADC map or T2 weighted images as reference. One ROI outlined the biopsy proven PCa lesion, that was previously described as PI-RADS 3, 4 or 5, the second ROI was placed in the mirror area in morphologically normal prostate tissue and the third ROI was placed in the obturator internus muscle. In the negative control group, the ROI was also placed in the morphologically normal peripheral or transitional zone and the obturator internus muscle. For every ROI, the above listed pharmacokinetic parameters were calculated and the ratio was obtained by dividing the values calculated in cancer and normal prostate tissue by the values calculated in the obturator internus muscle.

We used muscle to calculate ratio values that we then compared. We calculated the tissue-to-muscle ratios (how many times K_trans_ in cancer is higher in relation to muscle, how many times K_trans_ in normal tissue is higher in relation to muscle…) and then made groups that we further compared. We compared parameters (K_trans_, K_ep_, iAUC, V_e_) always in two groups (group 1—normal tissue in negative control group and normal tissue in morphologically normal tissue in prostate with cancer; group 2—normal tissue and cancer; group 3—cancer in peripheral zone and cancer in transitional zone).

The same parameter from one group was compared with the same parameter from the other group (K_trans_ from one group with K_trans_ from other group; K_ep_ from one group with K_ep_ from the other group; iAUC from one group with iAUC from the other group; V_e_ from one group with V_e_ from the other group).

Histopathology reports and laboratory values (prostate specific antigen, PSA) were obtained from the hospital administration system.

All patients were in the MRI scanner for an hour before the DCE study, so the exercise perfusion effect on muscles is assumed to be minimal.

### 2.4. Statistical Analysis

To compare quantitative data on pharmacokinetic parameters (K_trans_, K_ep_, iAUC and V_e_ value) and the difference of statistical significance concerning the tumour location (peripheral or transitional zone), the nonparametric Mann-Whitney U-test was used. The significance level was set to 0.05.

The statistical dependence between variables was assessed using Spearman’s rank correlation coefficient.

Logistic regression was used for analysing the possibility of using individual variables in the identification of diseased tissue and for evaluating the diagnostic performance of pharmacokinetic parameters (K_trans_, K_ep_, iAUC, V_e_) in differentiating prostate malignancy from normal prostate tissue, as well as for selecting the most predictive model based on these parameters.

Common indicators such as the Akaike Information Criterion (AIC) and the area under the ROC curve (AUC) were used to assess the quality of the model. Sensitivity, specificity and accuracy were calculated according to the formula:

Sensitivity = TP/(TP + FN) = (Number of true positive assessment)/(Number of all positive assessment); Specificity = TN/(TN + FP) = (Number of true negative assessment)/(Number of all negative assessment); Accuracy = (TN + TP)/(TN + TP + FN + FP) = (Number of correct assessments)/Number of all assessments)

Specificity and sensitivity were calculated for every cut-off value to select the appropriate value for the desired sensitivity.

In addition, the optimal cut-off value was determined using the Youden index.

## 3. Results

### 3.1. Patients and Lesion Characteristics

The mean age of the patients was 67 (age range from 42 to 83). Mean PSA levels were 14.47 ng/mL (range from 1.64 to 64.3). The total number of patients with biopsy proven PCa after mpMRI was 56, with a total number of lesions 70; 39 of them located in the peripheral zone, 31 in the transition zone (10 patients with Gleason score 4 + 3 = 7, 28 with Gleason score 3 + 4 = 7, 27 with Gleason score 3 + 3 = 6, 4 with Gleason score 4 + 4 = 8 and 1 patient with Gleason score 5 + 3 = 8). The total number of patients in the negative control group was 54.

### 3.2. Descriptive Statistics of Quantitative Pharmacokinetic Parameters in Cancer-to-Muscle Ratio and Normal Tissue-to-Muscle Ratio

First, we calculated ratios of values in cancer and normal tissue with values in muscle (group 1: normal tissue-to-muscle ratio in negative control group and normal tissue-to-muscle ratio in mirror area of morphologically normal prostate tissue with detected organ confined unilateral lesions; group 2: normal tissue-to-muscle ratio and cancer-to-muscle ratio; group 3: transitional zone cancer-to-muscle ratio and peripheral zone cancer-to-muscle ratio). Then, we compared these ratios in every group.

Differences in variables between normal tissue in the negative control group and mirror area in morphologically normal prostate tissue with detected organ confined unilateral lesions were tested using the Mann-Whitney U-test and proved that there was no statistically significant difference in K_trans_ (*p* = 0.896), K_ep_ (*p* = 0.828), iAUC (*p* = 0.340) and V_e_ (*p* = 0.679) values (Table 1).

The differences in variables for normal tissue and carcinoma were tested using the Mann-Whitney U-test and proved to be significant in K_trans_, K_ep_ and iAUC, but in V_e_ there was no statistically significant difference (Table 2).

The Mann-Whitney U-test did not confirm statistically significant differences in quantitative pharmacokinetic parameter ratio values between the peripheral and transitional carcinoma zone (Table 3).

All quantitative parameters are in positive correlation except K_ep_ and V_e,_ where there was no statistically significant monotone connection. Spearman’s rho coefficient for K_trans_ and K_ep_ was 0.558 (*p* < 0.001), for K_trans_ and iAUC 0.782 (*p* < 0.001), for K_trans_ and V_e_ 0.395 (*p* < 0.001), for K_ep_ and iAUC 0.609 (*p* < 0.001), for K_ep_ and V_e_ 0.037 (*p* = 0.662) and for iAUC and V_e_ 0.429 (*p* < 0.001).

### 3.3. Predicting Prostate Carcinoma

Logistic regression was used to assess the possibility of using individual variables in the identification of cancer. K_trans_ has the best predictive value on its own (AIC = 129; AUC = 0.892), then iAUC (AIC = 141; AUC = 0.843) followed by K_ep_ (AIC = 183; AUC = 0.786). V_e_ alone does not have any significant predictive value in the identification of prostate cancer (AIC = 193; AUC = 0.596).

After that we used the Youden index to determine the cut-off value for individual variables in determining cancer. According to the Youden index, the cut-off value for K_trans_ was 0.3048456 (specificity 72.85%; sensitivity 91.43%), for K_ep_ 0.4112993 (specificity 54.28%; sensitivity 92.86%) and for iAUC 0.4216619 (specificity75.72%; sensitivity 82.86%).

After determining the cut-off value, we wanted to express the cut-off value obtained using the Younden index in the value of K_trans_ increase in detected lesion in relation to K_trans_ value in muscle. In other words, we wanted to know how many times the K_trans_ value of the lesion must be higher than the K_trans_ value in the muscle in order to suggest cancer.

The cut-off value for K_trans_ expressed using the Younden index can be translated into a variable value using the obtained model Ln(θ/(1 − θ)) = −3.046 + 0.559 K_trans_, that is K_trans_ = (1/0.559)( Ln(θ/(1 − θ)) + 3.046) where θ is the calculated cut-off value.

Using this model, for θ value 0.3048456, the marginal cut-off value of K_trans_ increase in the lesion relative to muscle for the differentiation between cancer and normal prostate tissue is 3.974368 (specificity 72.86%; sensitivity 91.43%).

This means when K_trans_ value is 3.974368 times higher in detected lesion compared to K_trans_ value in muscle we can suggest cancer with 72.86% specificity and 91.43% sensitivity.

After assessing the possibility of using individual variables in the identification of cancer, we assessed the possibility of creating a model by combining the two parameters with the best individual predictive value.

To create a model with a higher predictive value for detecting cancer, K_trans_ and iAUC were also combined using logistic regression. This model with both variables demonstrated AIC 125, AUC 0.900, for a generated cut-off value of 0.438, with accuracy of 84.3%, specificity 85.7% and sensitivity 82.9%. A more detailed presentation of the parameter estimate is given in Table 4.

Using this model, the different cut-off values were calculated in terms of scores (Score = −3.505 + 0.413 K_trans_ Ratio + 0.245 iAUC Ratio) (Supplementary Materials Table S1).

According to the Youden index the suggested cut-off value was −0.2996502 with specificity 81.43% and sensitivity 87.14%. This means that, using this model, 12.8% of prostate carcinoma could remain undetected. If we use a cut-off value of −1.19002, we can expect to detect 100% of carcinoma. Given that the specificity is then 64.28%, we can also expect 35.7% false positive findings. Considering a cut-off of 2.701215 with 100% specificity, we would have sensitivity of 37.14% which means that 62.8% of prostate cancers could be missed.

## 4. Discussion

Solid tumours like PCa are characterized by angiogenesis, with new vessels being highly permeable, irregular and thin [29]. This, along with higher micro-vessel density, is proven to be associated with a higher Gleason score and metastatic potential [15,30]. DCE MRI enables the measurement of contrast agent distribution, providing information on tumour microcirculation and capillary permeability [31]. This has a role not only in locoregional staging of the disease but also in monitoring of oncologic treatment response [31]. Therefore, there is a great scientific interest in researching the increasing usability of DCE obtained pharmacokinetic parameters.

In our study, the values of K_trans_, K_ep_ and iAUC were significantly higher in cancer with a Gleason score ≥ 3 + 3 = 6, compared to normal prostate tissue. This suggests an increased permeability in cancer, which is consistent with previous studies [23,32,33,34,35,36]. V_e_ was not significantly higher, which is also consistent with previous studies [35,36].

Oto A. et al. found that in the central gland K_trans_ used together with ADC values improves the differentiation between central gland cancer, stromal and glandular hyperplasia [37]. Ocak I. et al. found that in the peripheral zone and the area under the gadolinium curve (AUGC), K_trans_, K_ep_ are significantly higher in prostate cancer compared to the normal peripheral zone, but V_e_ did not vary for prostate cancer and the normal peripheral zone [38]. Bonekamp D. and al. concluded that quantitative DCE parameters have a direct connection to pathophysiologic properties of prostatic tissue with an increased K_trans_ value that represents a mixed effect of increased both the permeability and flow due to tumour neoangiogenesis and a decreased V_e_, which represents the extravascular extracellular space, because new vessels occupy more volume compared to normal tissue, but with less specificity because V_e_ can also be decreased in benign prostatic hyperplasia [39].

Regarding the extravascular extracellular space volume in cancer, multiple studies have had different findings. Sanz-Requena R. et al. have found a statistically significant difference between V_e_ values in prostate carcinoma and prostate healthy tissue [40]. Jackson A.S. et al. had similar results [41], as well as Ludemann L. et al. [42]. Unlike them, Chen Y.I. [43] and Kozlowski P. [44] did not find a statistically significant difference between V_e_ values in prostate carcinoma and healthy prostate tissue.

Studies that support reduced V_e_ values in prostate cancer explain it with hypercellularity, which is a theory supported by research that studied V_e_ changes in response to oncologic therapy [45,46,47,48]. However, V_e_ is not only influenced by vessel and cell density, but also interstitial fluid pressure. It reflects complex relationships influenced not only with extravasation from intravascular space, but also with a disturbed ration of hydrostatic and osmotic pressure at the level of each cell within the lesion due to changes in the nucleus, stress induced collapse of lymphatic vessels and a secretion of various factors that induce angiogenesis or matrix degrading enzymes [49]. This could explain the varying V_e_ values.

A very promising finding in other research is that pharmacokinetic parameters obtained in DCE study correlate with the clinical stage or Gleason score. Wu X. et al. found in their study that K_trans_, K_ep_ and iAUC were higher in the clinical stage T2 than in the lower stage, and also that serum PSA correlates with K_trans_ and iAUC [50]. Wei C. et al. found K_trans_ and K_ep_ significantly different in prostate cancer with a Gleason score ≥ 3 + 3 = 6 compared to prostate tissue with benign changes and suggested the cut-off values for K_trans_ 0.205/min and K_ep_ 0.625/min [51]. Cristel G. et al. found that K_trans_ and K_ep_ values were significantly higher in prostate cancers with a Gleason score ≥7, compared to prostate cancers with a Gleason score <7 and suggested a K_trans_ cut-off value 191 × 10^−3^/min with accuracy 0.87, sensitivity 0.95 and specificity 0.61. However, they could not suggest a clinically acceptable cut-off value for K_ep_ with an accuracy above 0.80 [23]. Similar findings were also discovered by Sanz-Requena R. et al. and Cho E. et al., who suggested a K_trans_ cut-off value 210 × 10^−3^/min and 184 × 10^−3^/min, respectively [40,52]. Peng Y. et al. suggested a cut-off value for K_trans_ 0.257/min [53].

The differences in the suggested cut-off values result from a difference in the used MRI scanners, MR imaging parameters, postprocessing software and mostly because of the selected patients group characteristics. Most of this research emphasised the need for better standardisation.

Von Niekerk et al. noticed in their study that there is a great interpatient variability for absolute values for cancer and for normal tissue due to various factors. They found a correlation between pharmacokinetic DCE MRI parameters and pathohistological micro-vessel parameters only when they used cancer-normal prostate ratio, but not when they used absolute values [54]. They suggested that using cancer-normal tissue ratio could correct interpatient variability in interpretation. Also, Sanz-Requena R. et al. found that pharmacokinetic parameters can be used to predict prostate cancer aggressiveness when the obtained values are correlated with values in the normal peripheral zone [40].

Finding morphologically normal prostate tissue can sometimes be a very challenging task. Also, although the negative predictive value of multiparametric magnetic resonance imaging of the prostate is very high, between 90–93%, there is still a 7–10% chance for false negative findings if we use morphologically normal prostate tissue as reference.

Unlike previous research, which suggested an absolute cut-off value for specific parameters, we have suggested a comparison of pharmacokinetic parameters in the detected lesion with pharmacokinetic parameters in the obturator internus muscle. Calculating lesion-to-muscle ratio could be useful in decreasing interpatient variability and parameter effect that influence the DCE study, such as blood flow characteristics or cardiac output.

If we use individual variables in the cancer identification, K_trans_ has the best predictive value on its own (AIC = 129; AUC = 0.892).

In our study we also suggested a model combining K_trans_ and iAUC ratio values as two parameters with the best individual predictive values. This model demonstrated a higher AUC and lower AIC compared to using individual variables (AIC 125, AUC 0.900).

Previous studies have demonstrated that muscles can be useful in comparison with tumours [55,56,57,58,59,60,61].

Muscles have slow perfusion compared to other tissues, they have less heterogenicity and studies have suggested that DCE MRI parameters in tumour tissue in reference with normal tissue like muscles can potentially compensate for the variations in factors that alter these parameters, like cardiac output, assuming that normal tissue has less kinetic variability [47,53,57,62]. Noworolski S. et al. found in their study that prostatic cancer had a greater initial enhancement slope, shorter peak time, greater peak enhancement compared to normal prostate tissue [63]. In order to reduce interpatient variability, they normalised the calculated parameters to the same parameters in muscles, which decreased the interpatient variability for the peak enhancement [54,63].

Our study has several limitations. Firstly, histopathology was obtained in different ways, mostly through targeted biopsy. Less than a quarter of patients had radical prostatectomy and TRUS prostate biopsy is characterized by sampling errors and sometimes underestimation of lesions.

Also, in institutions where MR/US fusion biopsy is not used, it could be hard for the urologist who performed biopsy to correlate with MR findings. Our results demonstrate a significant difference between PCa and normal tissue which speaks in favour of accurate locating.

Taking into account the total sample size, the base is not very large and for this reason the validation of the model was carried out by cross validation and not by dividing the sample into a training and testing part. A division in this way would significantly impoverish the quality of the assessment and does not contribute to the assessment of fit measures on the testing sample. In addition, we have a number of statistical ways to control the quality of the model, such as AIC, deviance and others.

We believe the results still have value. They are certainly indicative because they show how predictive the model can be and that it can help in directing further research that is needed to refine this model.

Regarding MR examinations and analysis, AIF is established using a population-based AIF, contrary to individualized AIF, but in clinical use it is considered to be justified [64].

There is also a need to perform an assessment of reliability of the ROI contouring between more radiologists with analysis of the Interclass Coefficient Correlation of the extracted parameters, but currently this was not feasible within our research team.

We are aware of the shortcomings of the model, but the intention was not to build a model that will be used in practice but to prove that a quality predictive model can be built on the basis of these values.

After these results, it is clear that we need to focus on K_trans_ and iAUC, look for new data, larger group of patients, and independent evaluators who will provide us with sufficient quality data to develop an applicable model.

Further studies are needed with larger group of patients and a wider range of Gleason scores to examine the possibility of determining the aggressiveness of the detected disease.

Also, further studies are needed to evaluate these findings and to reach a higher specificity while maintaining high sensitivity in order to have higher true negative and lower false positive rate, and to reduce the number of unnecessary biopsies.

To our knowledge, this is the first study that compares quantitative parameters in prostate cancer, healthy tissue and muscle, with proposed cut-off values in terms of cancer-to-muscle ratios, while proposing a model which uses a combination of two parameters.

## 5. Conclusions

In conclusion, DCE MRI is a non-invasive tool for the assessment of prostate cancer neoangiogenesis and is complementary with T2 and DWI sequences in detecting prostate cancer by providing information about the tissue perfusion which can help direct diagnosis, treatment choice or treatment response. The use of quantitative DCE pharmacokinetic parameters compared to the obturator internus muscle as a reference tissue leads to higher diagnostic accuracy for detecting prostate cancer. In our study, the values of K_trans_, K_ep_ and iAUC were significantly higher in cancer with a Gleason score ≥ 3 + 3 = 6 compared to normal prostate tissue.

We determined an increase in the K_trans_ value in the lesion-to-muscle ratio with a cut-off value of 3.974368 for differentiating between prostate cancer and normal tissue with specificity of 72.86% and sensitivity of 91.43%.

We have also suggested combining K_trans_ and iAUC in a model that demonstrated higher AUC and lower AIC compared to using individual variables.

Based on the calculated quantitative pharmacokinetic parameters we suggested a threshold that provides 100% detection of prostate cancer, with 35.7% false positive detections.

## Figures and Tables

**Table 1:** Quantitative pharmacokinetic parameters in normal-to-muscle ratio of the negative control group categorized PIRADS 1 or 2 (NC) and normal-to-muscle ratio in the mirror area morphologically normal prostate tissue with detected organ confined unilateral lesions (N).

	ROI	K_trans_	iAUC	V_e_	K_ep_
Number	N	70	70	70	70
	NC	54	54	54	54
Mean	N	3.66	3.14	3.53	1.51
	NC	3.64	3.37	3.31	1.65
Median	N	3.14	3.04	2.93	0.98
	NC	3.26	3.37	3.09	1.1
Standard deviation	N	2.2	1.8	2.09	1.74
	NC	2.07	1.43	1.92	2.71
Minimum	N	1.19	0.185	0.419	0.152
	NC	1.15	0.771	0.404	0.167
Maximum	N	11.2	9.02	11.8	10.7
	NC	11.5	7.45	11.7	19.4
25th percentile	N	2.01	1.79	1.97	0.696
	NC	2.34	2.42	2.08	0.688
50th percentile	N	3.14	3.04	2.93	0.98
	NC	3.26	3.37	3.09	1.1
75th percentile	N	4.58	4.39	4.48	1.52
	NC	4.23	4.1	3.79	1.56

**Table 2:** Quantitative pharmacokinetic parameters in carcinoma-to-muscle ratio and normal tissue-to-muscle ratio.

	Mean		Median		Standard Deviation		Min		Max		*p*-Value
**ROI**	Normal	Pca	Normal	Pca	Normal	Pca	Normal	Pca	Normal	Pca	
**K_trans_**	3.63	9.83	3.06	7.14	2.17	6.4	1.19	3.19	11.2	28.8	1.226 × 10^−15^
**K_ep_**	1.51	3.54	0.98	2.01	1.74	5.27	0.152	0.319	10.7	31.1	5.192 × 10^−9^
**iAUC**	3.53	8.26	2.93	6.84	2.09	4.98	0.419	1.06	11.8	25.6	2.686 × 10^−12^
**V_e_**	3.14	3.96	3.04	3.58	1.8	2.37	0.185	0.4	9.02	11.2	0.05088

**Table 3:** Quantitative pharmacokinetic parameter ratio values in the peripheral (PZ) and transitional carcinoma zone (TZ) (min-minimal value; sd-standard deviation).

		PZ	TZ	*p*-Value
**K_trans_**	min	3.189	3.373	0.4307
	median	6.68	9.18	
	mean	8.973	10.903	
	sd	5.873	6.952	
	max	28.786	28.524	
**K_ep_**	min	0.5964	0.319	0.4238
	median	1.9764	2.045	
	mean	2.9023	4.35	
	sd	4.098	6.437	
	max	25.8947	31.123	
**iAUC**	min	1.057	1.162	0.1988
	median	6.25	7.277	
	mean	7.66	9.022	
	sd	4.716	5.281	
	max	22	25.581	
**V_e_**	min	0.4	0.853	0.4854
	median	3.446	3.581	
	mean	3.836	4.114	
	sd	2.502	2.225	
	max	10.517	11.236	

**Table 4:** Presentation of parameter estimate in model.

**Model Fit Measures**			
**Model**	**Deviance**	**AIC**	**R^2^_McF_**
1	119	125	0.385
**Model Coefficients—Ca**			
**Predictor**	**Estimate**	**SE**	***p***
Intercept	−3.505	0.627	<0.001
Ktrans	0.413	0.117	<0.001
iAUC	0.245	0.114	0.032

Note. Estimates represent the log odds of “Ca = 1” vs. “Ca = 0”.

## Data Availability

Not applicable.

## Supplementary Materials

The following supporting information can be downloaded at: https://www.mdpi.com/article/10.3390/tomography8030124/s1, Table S1: Cut-off values in terms of scores.
