# Supplementary material for: Dynamic Contrast Enhanced Study in Multiparametric Examination of the Prostate—Can We Make Better Use of It?
Source: Tomography. 2022 Jun 9;8(3):1509–21. doi: 10.3390/tomography8030124 (PMC9231365; doi:10.3390/tomography8030124)
Supplement: Supplementary file 1 [file tomography-08-00124-s001.zip › tomography-1637180-supplementary.pdf]

threshold	specificity	sensitivity	threshold	specificity	sensitivity	threshold	specificity	sensitivity
-Inf	0	1	-1.02836	0.657143	0.928571	0.790096	0.885714	0.514286
-2.66082	0.014286	1	-0.99241	0.671429	0.928571	0.88953	0.9	0.514286
-2.60261	0.028571	1	-0.9603	0.671429	0.914286	1.068.181	0.9	0.5
-2.54941	0.057143	1	-0.93245	0.671429	0.9	1.255.032	0.9	0.485714
-2.49298	0.071429	1	-0.88728	0.685714	0.9	1.44787	0.914286	0.485714
-2.45695	0.085714	1	-0.82679	0.7	0.9	1.608311	0.914286	0.471429
-2.41877	0.1	1	-0.80529	0.714286	0.9	1.675484	0.928571	0.471429
-2.40226	0.128571	1	-0.73248	0.714286	0.885714	1.707437	0.942857	0.471429
-2.36022	0.142857	1	-0.63989	0.728571	0.885714	1.767669	0.942857	0.457143
-2.29757	0.171429	1	-0.50382	0.742857	0.885714	1.826922	0.957143	0.457143
-2.26143	0.185714	1	-0.38712	0.757143	0.885714	1.848011	0.957143	0.442857
-2.22523	0.2	1	-0.38122	0.757143	0.871429	1.853801	0.971429	0.442857
-218.518	0.214286	1	-0.36084	0.771429	0.871429	1.889338	0.971429	0.428571
-2.16566	0.228571	1	-0.32833	0.785714	0.871429	1.924979	0.971429	0.414286
-2.15905	0.242857	1	-0.31344	0.8	0.871429	1.971379	0.985714	0.414286
-2.12335	0.257143	1	-0.29965	0.814286	0.871429	2.158728	0.985714	0.4
-2.09039	0.271429	1	-0.27972	0.814286	0.857143	2.429778	0.985714	0.385714
-208.069	0.285714	1	-0.25624	0.828571	0.857143	2.593167	0.985714	0.371429
-2.04931	0.3	1	-0.22884	0.828571	0.842857	2.701215	1	0.371429
-1.94698	0.314286	1	-0.21765	0.828571	0.828571	2.788138	1	0.357143
-1.85676	0.328571	1	-0.21384	0.828571	0.814286	2.860594	1	0.342857
-1.82167	0.342857	1	-0.15951	0.828571	0.8	3.157402	1	0.328571
-1.79188	0.357143	1	-0.10085	0.828571	0.785714	3.564984	1	0.314286
-1.77311	0.371429	1	-0.08079	0.828571	0.771429	3.838291	1	0.3
-1.76021	0.385714	1	-0.0595	0.828571	0.757143	4.049049	1	0.285714
-1.7595	0.4	1	0.006473	0.842857	0.757143	4.211528	1	0.271429
-1.73558	0.414286	1	0.07838	0.857143	0.757143	4.403541	1	0.257143
-1.61963	0.442857	1	0.134185	0.857143	0.742857	4.616431	1	0.242857
-1.52305	0.471429	1	0.1768	0.857143	0.728571	4.718711	1	0.228571
-1.48687	0.485714	1	0.179736	0.857143	0.714286	4.829765	1	0.214286
-1.44573	0.5	1	0.188782	0.857143	0.7	4.93425	1	0.2
-1.42846	0.514286	1	0.213936	0.871429	0.7	5.067708	1	0.185714
-1.41623	0.528571	1	0.253011	0.871429	0.685714	5.429023	1	0.171429
-1.40205	0.542857	1	0.275726	0.871429	0.671429	5.84023	1	0.157143
-1.37022	0.557143	1	0.301966	0.871429	0.657143	6.359393	1	0.142857
-1.31931	0.571429	1	0.340052	0.871429	0.642857	7.11724	1	0.128571
-1.25527	0.585714	1	0.373936	0.871429	0.628571	8.030403	1	0.114286
-1.21835	0.6	1	0.420005	0.871429	0.614286	8.563157	1	0.1
-1.20735	0.628571	1	0.464353	0.885714	0.614286	8.64366	1	0.085714
-1.19002	0.642857	1	0.492963	0.885714	0.6	9.551331	1	0.071429
-1.17999	0.642857	0.985714	0.518181	0.885714	0.585714	10.43716	1	0.057143
-1.17752	0.642857	0.971429	0.552225	0.885714	0.571429	10.64752	1	0.042857
-1.1647	0.642857	0.957143	0.593165	0.885714	0.557143	1124672	1	0.028571
-1.10687	0.642857	0.942857	0.674497	0.885714	0.542857	12.71733	1	0.014286
-1.05925	0.657143	0.942857	0.750178	0.885714	0.528571	Inf	1	0

Table S1. Cut-off values in terms of scores
